# Society Meetings

**Published:** 1916-11

**Authors:** 


### SOCIETY MEETINGS

Brief reports and announcements of meetings of societies of Western New York. and those of general scope, are requested from Secretaries. Copy should be on hand the fifteenth of the month. Full transactions will be published at cost of composition.

---

**DOCTOR: Is your Society properly represented here? If not, please bring our standing announcement to the attention of the members.**

---

The regular meeting of the **Medical Society of the County of Erie** was held October 16th, 1916, at 8:45 p. m. in the Buffalo Medical College. An unusually large number of members were present to hear the addresses of officers of the Medical Society of the State of New York by Dr. Martin B. Tinker, Dr. Floyd M. Crandall and James Taylor Lewis, Esq., President, Secretary and Attorney for the State Society respectively. Dr. M. N. Brown of Rochester, President of the Seventh District Branch was likewise present as a guest of the Society and delivered a brief address.

It was decidedly a State Society meeting, all the addresses were eloquent and to the point each one treating his subject in an admirable manner and commanding the closest attention of every member present.

At the conclusion of the addresses a hearty vote of thanks was tendered to all the speakers of the evening, who had come a long distance in order to become personally acquainted with the Society.

Nominations of officers for the ensuing year to be voted upon at the annual meeting in December were made as follows:

President, Dr. Irving W. Potter.

First Vice-President, Dr. George M. Cott.

Second Vice-President, Dr. James E. King.

Secretary, Dr. Franklin C. Gram.

Treasurer, Dr. Albert T. Lytle.

Censors, Dr. John D. Bonnar, Dr. Francis E. Fronczak, Dr. Arthur G. Bennett, Dr. A. D. Carpenter, Dr. Frank A. Valente.

Chairman Committee on Legislation, Dr. Harvey R. Gaylord.

Chairman Committee on Public Health, Dr. Nelson G. Bissell.

Chairman Committee on Membership, Dr. William F. Jacobs.

Chairman Committee on Economics, Dr. John V. Woodruff.

Delegates to the State Society, Dr. Charles G. Stockton, Dr. Irving W. Potter, Dr. A. W. Hurd, Dr. E. L. Frost, Dr. Edith R. Hatch, Dr. Grover W. Wende, Dr. Henry R. Hopkins, Dr. A. J. Colton, Dr. S. A. Dunham.

The following 37 candidates were elected to membership: Reinstatement of Dr. E. A. D. Clark and Dr. F. G. Walz. Elected to membership: Drs. O. Von Renner, J. Heller, A. L. Barone, A. W. Thompson, G. E. Stanbro, W. R. Coakley, J. Kiebala, F. A. Hayes, J. J. Conlon, J. F. Healy, F. H. Long, C. C. Panzarella, M. E. Bork, H. B. Johnson, C. E. Pringle, V. Reinstein, S. H. Levy, E. H. Lormor, H. Fairbanks, H. Muscato, A. H. Aaron, H. C. Hoffman, A. Burkel, L. C. Sampson, R. DeGraff, R. E. Shaver, P. S. Pearsons, A. F. Glaser, H. K. Hardy, A. H. Vogt, A. Horwitz, W. O. Hill, J. Brumberg, R. C. Miller.

Dr. Grover W. Wende offered the following resolution, which was referred to the Council:

Resolved: That the Medical Society of the County of Erie believes that the matter of defense from malpractice suits is of vital importance to the various members of the State Society and that while that defense has until the present time been conducted in a most satisfactory manner, a wise forethought should lead us to provide for contingencies which may reasonably be anticipated. We believe that additional counsel should be employed and trained in this highly special form of legal practice, and further

That inasmuch as the present revenues of the State Society are not sufficient to provide for this greatly needed progress we believe that the annual dues of the members of the State Society should be increased to four dollars per year.

On behalf of Dr. Lucien Howe, who was unable to be present, Dr. John V. Woodruff presented the following resolution, which was also referred to the Council:

Resolved: That the Committee on Economics be requested to report at its next meeting on the possibility of providing small but substantial relief to physicians who have been members in good standing of this Society for more than fifteen years, and who have become incapacitated from active work because of age, illness or other causes.

Moreover, if such assistance seems possible, the Committee is hereby directed to present a plan to provide for this in accordance with the resources of the Society.

Dr. Benedict invited all the members of the Society to attend the annual meeting of the Central New York Medical Society, which was to be held in Buffalo on October 19th, 1916.

Dr. Benedict offered the following resolution, which was seconded by Dr. Wende and carried.

Resolved: The Secretary be empowered to issue certificates to such members who wish to join the Central Medical Society of New York.

The Society then adjourned after which a collation was served in the College Library.

FRANKLIN C. GRAM,
Secretary.

---

47th Annual Meeting **American Medical Editors' Association,** New York City, Oct. 25 and 26. Annual banquet Thursday evening. Literary program:

President's Address, Edward C. Register, M. D.

"Latin and Greek as Prerequisites of the Study of Medicine," A. Jacobi, M. D. (By invitation).

"The Responsibility of American Journalism," Geo. M. Piersol, M. D. (American Journal of Medical Sciences).

"The Indexing of Scientific Journals," Anna L. von der Osten. (By invitation). (Rockefeller Institute for Medical Research).

"Book Reviews in Medical Journals," H. S. Baketel, M. D. (Medical Times).

"Editorial Individuality," Ira S. Wile, M. D. (American Medicine).

"The Special Journal Versus—The General Medical Journal,'' William Benham Snow, M. D. (Journal of Electro Therapeutics and Radiology).

''The Editor's Prerogative in Editing Original Contributions,'' H. Edwin Lewis, M. D. (American Medicine).

''The Medical Journals and the Materia Medica,'' Francis Edward Stewart, M. D. (Practical Druggist).

''The Relationship between Medical Journals of the Day,'' W. A. Jones, M. D. (Journal-Lancet).

''The Medical Journal and its Sphere in Medical Progress,'' A. S. Burdick, M. D. (American Journal Clinical Medicine).

''Independence in Medical Journalism,'' Llewellin Eliot, M. D. (Va. Medical Semi Monthly).

''The Function of a State Medical Association Journal,'' Geo. W. Kosmak, M. D. (Bulletin Lying-in-Hospital).

''Editorial Collaborator,'' Samuel F. Brothers, M. D., Brooklyn, N. Y.

''Some Suggestions as to Practical Details of Medical Journalism,'' A. L. Benedict, M. D. (Buffalo Medical Journal).

Special Symposium. ''The duty of the medical editor in harmonizing the doctor with Medical Legislation and its administration, and in securing his aid, co-operation and support in framing and enforcing proper laws, with especial reference to anti narcotic legislation,'' Hon. C. M. Collins, (Judge of the Court of Special Sessions); Hon. John F. Freschi, (Judge of the Court of Special Sessions); Mr. B. C. Keith, (Chief of the Miscellaneous Division of Internal Revenue Bureau, Washington, D. C.); Prof. Reynolds Webb Wilcox, (Chairman of the Narcotic Legislative Committee. Society of Medical Jurisprudence); Dr. John D. Davin, (Representative of the Federation of Medical Economic Leagues); Mr. Burdett G. Lewis, (Commissioner of Corrections of the City of N. Y.); Ernest S. Bishop, A. B., M. D., (Professor Clinical Medicine, N. Y. Polyclinic and President of the Medical Board of the Dept. of Corrections of the City of N. Y.)

Officers, 1915-16: President, Edward C. Register, M. D., Charlotte, N. C., (Editor, Charlotte Medical Journal). First Vice-President, W. A. Jones, M. D., Minneapolis, Minn., (Editor, Journal-Lancet). Second Vice-President, G. M. Piersol, M. D., Philadelphia, Pa., (Editor, American Journal of Medical Sciences). Secretary and Treasurer, J. MacDonald, Jr., M. D., New York, (Mgr. Editor, American Journal of Surgery).

Executive Committee: Dr. C. F. Taylor, Philadelphia, (Medical World); Dr. John C. MacEvitt, New York, N. Y., (N. Y. State Journal of Medicine); Dr. A. S. Burdick, Chicago, Ill., (American Journal Clinical Medicine); Dr. J. MacDonald, Jr., New York, (American Journal of Surgery).

Publication Committee: Dr. H. Edwin Lewis, Chairman and Editor, New York, (American Medicine); Dr. C. E. de M. Sajous, Philadelphia, Pa., (Editor N. Y. Medical Journal); Dr. Chas. Wood Fassett, Kansas City, Mo., (Editor, Medical Herald); Dr. Robert M. Green, Boston, Mass., (Editor, Boston Medical Journal); Dr. J. MacDonald, Jr., Secretary, New York, (American Journal of Surgery).

Local Committee: Dr. Thomas L. Stedman, Chairman, New York, (Medical Record); Dr. R. H. Sayre, New York, (New York Medical Journal); Dr. Brooks H. Wells, New York, (American Journal Obstetrics); Dr. Frank C. Lewis, New York, (International Journal Surgery); Dr. Ira S. Wile, New York, (American Medicine).

---

**The Elmira Academy of Medicine** held its regular meeting Oct. 4. Dr. R. G. Loop gave a paper on Specific Immunization against Tuberculosis and Dr. B. G. Voorhes on Headache. Officers: President, LaRue Colegrove; Secretary, E. T. Bush.

---

**The Medical Society of the County of Wyoming** met in Warsaw, Oct. 10. The following officers were elected: President, L. H. Humphrey, Silver Springs; Vice-President, L. E. Stage, Bliss; Secretary and Treasurer, J. F. Crawford, Warsaw.

---

**The Rochester Academy of Medicine,** Section II, met Oct. 11. A symposium on Drainage vs. Removal of the Gall Bladder was presented by E. W. Mulligan, F. W. Zimmer, J. W. Magill, C. R. Barber, H. T. Williams, and W. B. Jones, with summing up by Donald Guthrie of Sayre, Pa. Officers: Chas. W. Hennington, Chairman; Wm. I. Dean, Secretary.

---

**The Medical Society of the County of Monroe** met Oct. 17. Henry H. Hazen of Washington gave an illustrated lecture on Skin Cancers. A dinner was given in his honor before the meeting. Chas. W. Hennington, Secretary.

---

**The Buffalo Academy of Medicine** meets weekly, except on special notice to the contrary, every Wednesday evening, in Orpheus Hall, excepting the last Wednesday in Dec., from Oct. to May inclusive. Out-of-town guests are welcome and should make their presence known to some member in order that they may be presented to the Academy and accorded the privilege of the floor. Residents of places outside of Buffalo but near enough so that frequent attendance is possible, may secure non-resident membership at a special rate. The

Academy meets, in rotation, as sections of Surgery, Medicine, Obstetrics including Gynaecology, and Pathology, all members being ipso facto members of all sections. The officers for the year 1916-17 are: Pres. Lawrence Hendee; Sec. Herbert A. Smith; Treas. B. F. Schreiner; Trustees, Earl P. Lothrop, Harry R. Trick and W. W. Plummer. Section of Medicine: Chairman Edward A. Sharp; Secretary Harry Mead. Surgery: Chairman Francis O'Gorman; Sec. James B. Cross. Obstetrics and Gynaecology: Chairman Irving W. Potter; Sec. H. K. De Groat. Pathology: Chairman Wm. F. Jacobs; Sec. Wm. G. Bissell. The Section Chairmen are vice-presidents of the Academy. The foregoing officers with the president for the preceding year, James W. Putnam, comprise the Council.

The Section of Surgery met Oct. 3, (Tuesday) when Dr. Joseph C. Bloodgood of Baltimore presented a lantern slide lecture on The Cancer Problem Based upon Recent Experiences.

The first stated meeting of the Academy was held Oct. 11, under the auspices of the Section of Medicine, Henry J. Mulford presenting a paper on Child Analysis. A collation was served at the close of the meeting.

The Section of Obstetrics and Gynaecology met Oct. 18. The program was as follows:

Analgesia and Anaesthesia in Obstetrics, Dr. John H. Evans. Discussion by Drs. Pearson, Getman and Trick.

Posterior Positions and Their Treatment, Dr. H. C. McDowell. Discussion by Drs. Goldsborough and W. G. Taylor.

Pregnancy in the Tubercular, Dr. E. A. D. Clarke. Discussion by Drs. Pryor and Geo. J. Eckel.

---

**Central New York Medical Association.** The 48th annual meeting of the Central N. Y. Medical Association was called to order by President A. L. Benedict at 10.35 a. m, on 10-19, '16, at the University Club, Buffalo, N. Y.

In the absence of the secretary, Dr. J. J. Buettner, Dr. Robert Burns of Syracuse was appointed secretary pro tem.

The minutes of the previous meeting were read and no objections thereto. President Benedict suggested that it appear in the minutes that there was no competition between the N. Y. State Journal and the Buffalo Medical Journal regarding the printing of the proceedings of the Society, but inasmuch as the N. Y. State Journal had expressed its inability to print them, the Buffalo Medical Journal had therefore been designated for the work.

The presidential address by Dr. A. L. Benedict was entitled "Highways." The president mentioned the policy of his predecessors in dwelling upon some general subject in their addresses, and in this one he referred both humorously and seriously to the condition of the roadways in N. Y. State.

In the absence of the treasurer, Dr. F. F. Laurie, the president appointed Drs. Edward Clark and O. W. Mead to audit the treasurer's report.

The first scientific paper was by Dr. C. W. Hennington of Rochester, N. Y., entitled "Gall bladder surgery, shall we drain or remove?"

The discussion was opened by Dr. Thew Wright of Buffalo. Remarks were made by Dr. W. A. Groat of Syracuse. The discussion was closed by Dr. Hennington.

The president called upon the credential committee, but as none of the members were present, he designated Drs. Crego, Wright and Jones to act pro tem.

The second scientific paper by Dr. H. A. Bray of Ray Brook was entitled "Some observations relative to the breath sounds in incipient tuberculosis." This paper was illustrated by lantern slide demonstrations by Dr. Frank H. Hunt of Ray Brook. Discussion on the topic was opened by Drs. J. N. Pryor, and Delancey Rochester of Buffalo. The discussion was closed by Dr. Bray.

Dr. Howe of Buffalo was granted the floor, and in a graceful speech he extended a cordial invitation to all members of the society, and especially out of town members, to be guests at a luncheon at his home at noon. The invitation was accepted.

The third scientific paper was on the general subject of "Infantile paralysis." This was divided into three parts—

(a) "Attitude of the family physician during the epidemic," by Dr. Irving M. Snow, Buffalo;

(b) "Differential diagnosis and treatment," by Dr. E. A. Shoop, Buffalo;

(c) "Surgical treatment," by Dr. W. W. Plummer, Buffalo.

The president ruled that discussions be limited to 10 minutes. Parts (a) and (b) of above were covered when adjournment was taken to luncheon at 1.05 p. m.

The afternoon session was opened with Pres. Benedict in the chair at 2.20 o'clock.

The general subject of "Infantile paralysis" was continued and part (c) was finished. The discussion of the general topic was then opened by Dr. Crego, with remarks by Drs. Rochester and Clark. Motion by Dr. Bartoo that Dr. Clark be granted an extension of time for discussion was seconded and carried. Discussion was closed by Drs. Sharp and Plummer.

Dr. Henry Buswell was granted the floor and reported some of the findings of the John Hopkins Pathological Society relative to this disease.

The fourth scientific paper by Dr. C. F. Hooper of Cleveland was entitled "The origin and distribution of bile pigment in the body and its diagnostic significance." The discussion was opened by Drs. Stockton, Buswell and Russell of Buffalo. Discussion closed by Dr. Hooper.

The fifth scientific paper by Dr. Dean Lewis of Chicago on "The hypophysis and its relation to general medicine," was illustrated by lantern slides by the essayist.

The sixth scientific paper by Dr. A. Stengel of Philadelphia was entitled "The diagnosis and some aspects of treatment of chronic nephritis."

Motion by Dr. Crego that discussion of above papers be omitted was seconded and carried.

The seventh scientific paper by Dr. Crego of Buffalo on "Treatment of disorders of sleep" was read by title.

The eighth scientific portion of the program by Dr. John M. Flint of New Haven, consisted of a talk on "Experiences at a base hospital in France. The speaker illustrated his remarks with lantern slide pictures.

The auditors, Drs. Edward Clark and O. M. Mead, reported treasurer's report correct.

Motion by Dr. Geo. F. Cott that a vote of thanks be extended by the association to the out of town essayists for their generous participation in the program, and for the interesting and instructive presentation of their respective subjects, was seconded and carried.

Motion by Dr. Clark of Skaneateles that the society express to Dr. and Mrs. Howe their hearty appreciation of the delightful entertainment afforded by them, and that the secretary be instructed to notify them in writing. Seconded and carried.

While at Dr. Howe's residence the members were addressed by Col. Kean, M. D., of the U. S. army on the need of establishing Red Cross Base Hospitals in various cities of the U. S.

The secretary pro tem. was directed by the president to convey to Secretary J. J. Buettner the deepest sympathy of the association on account of the serious illness of his wife.

BUSINESS SESSION

Motion by Dr. Crego that Dr. L. Heazlitt of Auburn be nominated for president of the society for the ensuing year. Seconded and carried.

Motion by Dr. Groat that the secretary cast an unanimous ballot for Dr. Heazlitt for president. Seconded and carried. The president ordered such ballot cast.

Motion by Dr. Breese of Syracuse that Dr. J. J. Buettner of Syracuse by nominated for first vice-president. Seconded and carried.

Motion by Dr. Groat that the secretary cast an unanimous ballot for Dr. Buettner for first vice-president. Seconded and carried. The president declared such ballot cast.

Motion by Dr. Crego that Dr. C. W. Hennington of Rochester be nominated for second vice-president. Seconded and carried.

Motion made by Dr. Price that the secretary cast an unanimous ballot for Dr. Hennington for second vice-president. Seconded and carried. The president reported such ballot cast.

Motion by Dr. Price that Dr. Robt. Burns of Syracuse be nominated for secretary for the ensuing year. Seconded and carried.

Motion by Dr. Clark of Skaneateles that the president cast an unanimous ballot for Dr. Burns for secretary. Seconded and carried. The president ordered such ballot cast.

Motion by Dr. Breese that Dr. Laurie of Auburn be nominated for treasurer for the ensuing year. Seconded and carried.

Motion made that the secretary cast an unanimous ballot for Dr. Laurie for treasurer. Seconded and carried. The president reported such ballot cast.

Motion by Dr. Crego that the treasurer pro tem., Dr. J. D. Bonnar of Buffalo be instructed to pay the lantern slide man $10 and the club steward $5. Seconded and carried.

The temporary membership committee reported names of Drs. J. D. Bonnar and Roland Meizenbach for members of the society.

Motion by Dr. Price that they both be elected to membership. Seconded and carried.

The president read a letter from the Auburn Academy of Medicine inviting the association to meet at Auburn next year, 1917.

Motion that the invitation be accepted. Seconded and carried.

Adjourned, 5.45 p. m.

ROBT. BURNS, Sec'y pro tem.

